# Supplementary material for: Insular Cell Integrity Markers Linked to Weight Concern in Anorexia Nervosa—An MR-Spectroscopy Study
Source: J Clin Med. 2020 Apr 30;9(5):1292. doi: 10.3390/jcm9051292 (PMC7288299; doi:10.3390/jcm9051292)
Supplement: Supplementary file 1 [file jcm-09-01292-s001.pdf]

Article

Insular cell integrity markers linked to weight concern in anorexia nervosa – An MR-spectroscopy study

S. Maier [1,2], K. Nickel [2], E. Perlov [2,3], A. Kukies [1], A. Zeeck [1], L. Tebartz van Elst [2], D. Endres [2], D. Spieler [1], L. Holovics [1], A. Hartmann [1], M. Dacko [4], T. Lange [4] and A. Joos [1,5]

1 Department of Psychosomatic Medicine and Psychotherapy, Medical Center – University of Freiburg, Faculty of Medicine, University of Freiburg, Germany
2 Department of Psychiatry and Psychotherapy, Medical Center – University of Freiburg, Faculty of Medicine, University of Freiburg, Germany
3 Luzerner Psychiatrie, Hospital St. Urban, St. Urban, Switzerland
4 Department of Radiology, Medical Physics, Medical Center – University of Freiburg, Faculty of Medicine, University of Freiburg, Germany
5 Department of Psychotherapeutic Neurology, Kliniken Schmieder, Germany
* Correspondence simon.maier@uniklinik-freiburg.de; Tel +xx-xxxx-xxx-xxxx

Supplementary table 1: Mutual correlation of MRS metabolites. The numbers indicate the correlation coefficient *r*.

	Cho	Ins	NAA	Glx
Ins	0.48			
NAA	0.54	0.40		
Glx	0.34	0.42	0.42	
Cre	0.54	0.48	0.56	0.51
